# Brentuximab vedotin-containing escalated BEACOPP variants for newly diagnosed advanced-stage classical Hodgkin lymphoma: follow-up analysis of a randomized phase II study from the German Hodgkin Study Group

**DOI:** 10.1038/s41375-021-01386-z

**Published:** 2021-08-18

**Authors:** Carla Damaschin, Helen Goergen, Stefanie Kreissl, Annette Plütschow, Frank Breywisch, Stephan Mathas, Julia Meissner, Martin Sökler, Max S. Topp, Vladan Vucinic, Andreas Zimmermann, Bastian von Tresckow, Michael Fuchs, Andreas Engert, Peter Borchmann, Dennis A. Eichenauer

**Affiliations:** 1 First Department of Internal Medicine, University of Cologne, Center for Integrated Oncology Aachen Bonn Cologne Dusseldorf, Cologne, Germany; 2 German Hodgkin Study Group (GHSG), University Hospital Cologne, Cologne, Germany; 3 Department of Hematology, Oncology and Palliative Care, Klinikum Ernst von Bergmann, Potsdam, Germany; 4 Department of Hematology, Oncology and Tumor Immunology, Charité-Universitätsmedizin Berlin, Max-Delbrück-Center for Molecular Medicine, Berlin, and Experimental and Clinical Research Center (ECRC), Berlin, Germany; 5 Fifth Department of Internal Medicine, University Hospital Heidelberg, Heidelberg, Germany; 6 Second Department of Internal Medicine, University Hospital Tübingen, Tübingen, Germany; 7 Second Department of Internal Medicine, University Hospital Würzburg, Würzburg, Germany; 8 Department of Hematology and Cell Therapy, Medical Oncology, Hemostaseology, University Hospital Leipzig, Leipzig, Germany; 9 Department of Internal Medicine III, University Hospital Munich, Munich, Germany; 10 Department of Hematology and Stem Cell Transplantation, West German Cancer Center, University Hospital Essen, University of Duisburg-Essen, Essen, Germany

**Keywords:** Phase II trials, Lymphoma, Combination drug therapy

Patients with advanced-stage Hodgkin lymphoma (HL) receiving intensive treatment with escalated BEACOPP (bleomycin, etoposide, doxorubicin, cyclophosphamide, vincristine, procarbazine, prednisone) (eBEACOPP) have excellent outcomes [1, 2]. However, late effects such as second primary malignancies (SPM) and infertility represent ongoing concerns [3, 4]. Novel approaches using an eBEACOPP backbone, therefore, aim at reducing toxicity without compromising efficacy. The BrECAPP (brentuximab vedotin, etoposide, cyclophosphamide, doxorubicin, procarbazine, prednisone) and BrECADD (brentuximab vedotin, etoposide, cyclophosphamide, doxorubicin, dacarbazine, dexamethasone) protocols were investigated in a randomized phase II study including patients with newly diagnosed advanced-stage classical HL (cHL). Both regimens combine eBEACOPP-based chemotherapy with the CD30-directed antibody-drug conjugate brentuximab vedotin (BV). Response rates were similar to standard eBEACOPP and especially the BrECADD protocol was associated with reduced acute toxicity [5].

The major objectives of the present follow-up analysis of the study were progression-free survival (PFS) and overall survival (OS) at 3 years. Patterns of cHL recurrence, the occurrence of SPM, and causes of death were also evaluated. PFS and OS were analyzed using the Kaplan–Meier method. PFS was defined as the time between randomization and progression or relapse of cHL or death from any cause and was censored at the date of last information on the disease status. OS was defined as the time between randomization and death from any cause and was censored at the date of last information for surviving patients. Characteristics of cHL recurrence, SPM, and causes of death were analyzed descriptively. Study design, inclusion criteria, endpoints, procedures, and treatment have been published elsewhere [5]. The study was conducted in accordance with the Declaration of Helsinki and was approved by the review boards of the participating sites. The study was registered at www.clinicaltrials.gov as #NCT01569204.

A total of 104 patients with advanced-stage cHL aged 18–60 years were enrolled at 20 sites in Germany. Baseline characteristics have been reported previously. In brief, 63 patients (61%) were male, the median age was 29 years (range: 18–60 years), and 86 patients (82%) presented with stage III/IV disease [5]. Baseline characteristics were thus consistent with the randomized German Hodgkin Study Group (GHSG) HD15 and HD18 studies for advanced-stage HL [1, 2]. Three patients were not included in the outcome analyses since they did not receive study treatment (*n* = 2) or terminated study treatment after the advanced-stage disease had been disconfirmed (*n* = 1). Hence, the present analysis included 101 patients who had been treated with 6 cycles of BrECAPP (*n* = 49) or BrECADD (*n* = 52); 13 patients (BrECAPP: 7 patients; BrECADD: 6 patients) with residual lymphoma ≥2.5 cm and a positive positron emission tomography (PET) at the end of systemic therapy had an indication for consolidation radiotherapy [5].

**Fig. 1:**
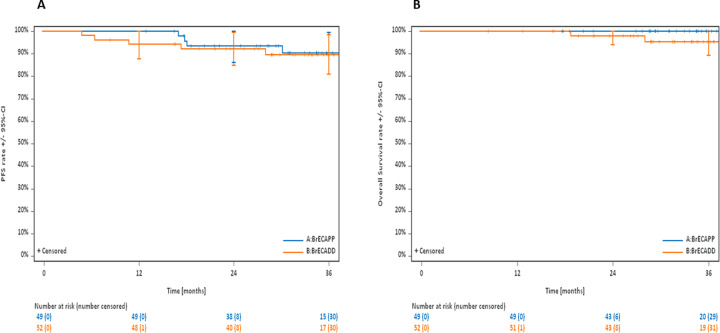
Outcomes of patients treated with BrECAPP and BrECADD. **A**) Progression-free survival after treatment with BrECAPP and BrECADD; **B**) Overall survival after treatment with BrECAPP and BrECADD.

After a median observation time of 34 months (interquartile range: 28.7–39.5 months), the 3-year PFS estimates were 90.2% (95%-CI: 80.9–99.5%) with BrECAPP and 89.7% (95%-CI: 81.0–98.3%) with BrECADD (Fig. 1A, Table 1). In the randomized ECHELON-1 study for newly diagnosed stage III/IV cHL, the 3-year PFS among patients aged younger than 60 years who had treatment with the BV-containing A-AVD (BV, doxorubicin, vinblastine, dacarbazine) regimen was 84.9% (95%-CI: 81.6–87.7%) [6]. Disease control with BrECAPP and BrECADD was thus at least similar to and possibly better than with A-AVD.

**Table 1:** Outcomes and events after BrECAPP and BrECADD treatment.

	6 × BrECAPP (*N* = 49)		6 × BrECADD (*N* = 52)	
Observation time				
Observation time for disease status, median	31 months		34 months	
Observation time for survival status, median	35 months		34 months	
3-year survival estimates				
Progression-free survival	90.2% (80.9–99.5%)		89.7% (81.0–98.3%)	
Overall survival	100%		95.4% (89.2–100%)	
Hodgkin lymphoma events^a^				
Any Hodgkin lymphoma event	4	(8%)	4	(8%)
Progression^b^	0		3	(6%)
Relapse^c^	4	(8%)	1	(2%)
Number of Hodgkin lymphoma events				
1	4	(8%)	2	(4%)
2	0		2	(4%)
Second-line treatment				
High-dose chemotherapy and autologous stem cell transplantation	4	(8%)	4	(8%)
Causes of death				
Any event	0		2	(4%)
Hodgkin lymphoma	0		1	(2%)
Accident	0		1	(2%)
Second primary malignancies				
Any event	0		0	

Data are median, % (95%-CI), or *n* (%). Only patients eligible for efficacy analyses are shown; 3 of 104 patients were excluded because they did not receive any study treatment (*n* = 2) or terminated treatment due to disconfirmation of advanced-stage disease (*n* = 1).

*BrECAPP* brentuximab vedotin, etoposide, cyclophosphamide, doxorubicin, procarbazine, prednisone, *BrECADD* brentuximab vedotin, etoposide, cyclophosphamide, doxorubicin, dacarbazine, dexamethasone.

^a^Defined as biopsy-proven disease progression or relapse of cHL.

^b^Defined as the occurrence of new lesions during treatment or at least 1 known lesion that increased by more than 25% in diameter during treatment or within 3 months after the end of systemic study treatment.

^c^Defined as the appearance of new lesions or the reappearance of initial lesions at least 3 months after the end of systemic study treatment.

Eight patients treated in the present study experienced primary disease progression (*n* = 3) or relapse of cHL (*n* = 5). Sites of initial bulky disease were involved in 6 cases. The median time from the end of BrECAPP or BrECADD therapy to disease progression or relapse was 13 months (range: 1–26 months). Second-line treatment consisted of high-dose chemotherapy and autologous stem cell transplantation in all cases. No study participant developed an SPM (Table 1). This is notable since older analyses had indicated an increased risk especially for the development of therapy-related acute myeloid leukemia and myelodysplastic syndrome (t-AML/MDS) after 8 cycles of eBEACOPP [3, 7, 8]. However, the current GHSG standard of care for advanced-stage HL consisting of 4 or 6 cycles of eBEACOPP stratified by PET after 2 cycles of chemotherapy appears to be associated with a significantly lower risk. The t-AML/MDS incidence among patients who had received 4 or 6 cycles of eBEACOPP within the HD15 and HD18 studies was lower than 1% [1, 2]. Unlike second hematologic malignancies that usually occur within the first years after HL treatment, the risk for the development of second solid tumors after BrECAPP and BrECADD cannot be estimated yet [7, 9, 10]. Longer follow-up is required since second solid tumors are typically diagnosed many years after HL treatment and the risk for the occurrence of such malignancies seems to be elevated for decades [11, 12].

Two patients who had treatment in the present study died during observation. Deaths occurred 15 and 24 months after the end of systemic study treatment. One of the deaths was due to cHL (Table 1). A low rate of HL-related deaths was also observed in other studies comprising patients with advanced-stage HL, e.g., the HD15 and HD18 studies using eBEACOPP and the RATHL study using ABVD [1, 2, 13].

The 3-year OS estimates were 100% with BrECAPP and 95.4% (95% CI: 89.2–100%) with BrECADD (Fig. 1B, Table 1). In the ECHELON-1 study, the 2-year OS among patients treated with A-AVD was 96.6% [14].

The present analysis has some limitations. Given the mostly young age at diagnosis and the high cure rate, quality of life (QoL) aspects and fertility issues play an important role in the management and the choice of treatment in HL patients [4, 15]. However, valid analyses on QoL and fertility in individuals who had received BrECAPP or BrECADD could not be performed due to the inability to obtain sufficient data.

Taken together, the present update analysis of a randomized phase II study investigating the BV-containing eBEACOPP variants BrECAPP and BrECADD in the first-line treatment of advanced-stage cHL confirms the safety and efficacy of these protocols. The BrECADD regimen had been chosen to challenge eBEACOPP in the randomized GHSG HD21 study (NCT02661503) that recently finished recruitment for the cohort of patients aged 60 years or younger. Results of this trial are pending. Results were in part presented at the EHA 2021 Virtual Congress, June 9 to 17, 2021.
